# Prediction of modifications in size and peri-infarct zone by T2-imaging after an acute myocardial infarction during longitudinal follow-up by cardiac MRI

**DOI:** 10.1186/1532-429X-13-S1-P150

**Published:** 2011-02-02

**Authors:** Damien Mandry, Bobak Heydari, Shuaib Abdullah, YiuCho Chung, Wolfgang Rehwald, Otavio R Coelho-Filho, François-Pierre Mongeon, Alanna S Harris, Rob J van der Geest, Michael Jerosch-Herold, Raymond Y Kwong

**Affiliations:** 1 Brigham and Women's Hospital, Boston, MA, USA; 2 Siemens Medical Systems, Chicago, IL, USA; 3 Leiden University Medical Center, Leiden, Netherlands

## Background

Fat suppressed T2-weighted black blood turbo spin echo (BBTSE) imaging can detect myocardial edema developed after acute myocardial infarction (MI) and characterize the extent of myocardium at risk in comparison to late gadolinium enhancement (LGE) imaging.

## Purpose

We hypothesized that T2-extent at baseline and changes during follow-up are associated with dynamic changes of infarct heterogeneity during the convalescence phase of infarct healing. We further explored the strength of association in quantifying T2-based myocardial edema, using a semi-automated method and manual tracing.

## Methods

Fifty-one patients (40 men) underwent 3T cardiac MRI both at 18±10 days and 7.7±1.1 months after an acute MI, including: 3 short-axis slices of T2-weighted fat-suppressed BBTSE, LV function assessment by SSFP and LGE imaging. Area of hyperintensity on T2 images was assessed by a semi-automated method using a threshold of mean signal intensity in the remote ROI ± 2 SD and by manual tracing. In further analyses, correlations of T2 extent, expressed as the percentage of total myocardium, and other MRI results were assessed by Spearman correlation.

## Results

Correlations between the T2 extent, LV parameters, infarct heterogeneity and their respective changes are displayed in the table. At baseline, hyperintense T2 myocardium was demonstrated in 50 patients (98%) and represented 20.5±14.0% of total myocardium. Baseline T2 size was inversely correlated with LVEF and was directly correlated with total MI size. Hyperintense T2 areas persisted in 12 patients (24%) at follow-up. Manual-tracing was correlated well with the semi-automated method (Spearman’s rho 0.84; *P*<0.0001). Table 1

**Table 1:** 

			Spearman’s coefficient	*P*
Extent of T2 at baseline	Baseline MRI	LVEF	-0.30	0.03

		LVEDVi	0.03	0.82

		LVESVi	0.15	0.30

		Total MI size	0.29	0.04

		PIZ size	0.30	0.03

	Changes in LV parameters and MI characteristics during follow-up	LVEF	-0.02	0.91

		LVEDVi	-0.03	0.84

		LVESVi	-0.07	0.62

		Total MI size	0.31	0.05

		PIZ size	0.41	<0.01

Changes in extent of T2	Changes in LV parameters and MI characteristics during follow-up	LVEF	-0.07	0.63

		LVEDVi	0.04	0.76

		LVESVi	0.03	0.86

		Total MI size	0.27	0.09

		PIZ size	0.33	0.04

Notes. T2 extent expressed as percentage of total myocardium (%). LV: left ventricle. EF: ejection fraction (%). EDVi: indexed end-diastolic volume (ml/m^2^). ESVi: indexed end-systolic volume (ml/m^2^). MI: Myocardial infarct. PIZ: peri-infarct zone.

## Conclusions

T2 extent early after an MI is a predictor of shrinkage of infarct size and regression of the PIZ.

While the clinical significance of persistent hyperintense T2 areas after initial infarct healing remains unknown, resolution of infarct edema by T2 imaging appears to be associated with a reduction of the periinfarct zone of LGE. Edema measurement by manual drawing was consistent with the 2SD threshold method and is a reliable quantitative method.

